# PRIMA-1^met^ (APR-246) inhibits growth of colorectal cancer cells with different p53 status through distinct mechanisms

**DOI:** 10.18632/oncotarget.5385

**Published:** 2015-10-01

**Authors:** Xiao-Lan Li, Jianbiao Zhou, Zit-Liang Chan, Jing-Yuan Chooi, Zhi-Rong Chen, Wee-Joo Chng

**Affiliations:** ^1^ Cancer Science Institute of Singapore, National University of Singapore, Centre for Translational Medicine, Singapore 117599, Republic of Singapore; ^2^ Department of Gastroenterology, Suzhou Municipal Hospital (Eastern), Suzhou City, Jiangsu Province, 215001, P.R. China; ^3^ Department of Medicine, Yong Loo Lin School of Medicine, National University of Singapore, Singapore 119074, Republic of Singapore; ^4^ Department of Hematology-Oncology, National University Hospital, Singapore 119228, Republic of Singapore

**Keywords:** colorectal cancer (CRC), p53, tumor suppressor gene (TSG), PRIMA-1^met^, targeted therapy

## Abstract

PRIMA-1^met^ (APR-246) is a methylated derivative and structural analog of PRIMA-1 (p53 re-activation and induction of massive apoptosis). PRIMA-1^met^ has been reported to restore both the wild type (wt) structure and function of mutant p53. Here, we show that PRIMA-1^met^ is highly effective at limiting the growth of CRC cells regardless of p53 status. However, PRIMA-1^met^ induces robust apoptosis only in CRC cells with mutant p53. Upregulation of Noxa, a proapoptotic molecule, is crucial for PRIMA-1^met^ mediated activity. In human xenograft model of disease, PRIMA-1^met^ effectively suppresses CRC tumor growth. Our results uncover distinct mechanisms of PRIMA-1^met^ in CRC with different p53 status, thus providing a mechanistic rationale to evaluate the clinical efficacy of PRIMA-1^met^ in CRC patients with different p53 status.

## INTRODUCTION

Colorectal cancer (CRC) is a heterogeneous disease with progressive accumulation of genetic and epigenetic aberrations and defects in immune surveillance [1–3]. Activation of oncogenes and inactivation/loss of tumor suppressor genes (TSGs) are crucial for transformation of malignant cells [4, 5]. p53, as a stress-inducible transcription factor, regulates a diversity of biological functions, such as cell cycle arrest, apoptosis, senescence, and autophagy, via mediating its plethora of target genes [6–8]. In CRC, p53 mutation occurs in about 40–50% of cases [9–11]. Mutated p53 loses its tumor suppressive function, which is a critical event in the adenoma to carcinoma transition during colon carcinogenesis [12]. Scientists have been enthusiastic in developing different strategies to reactivate mutated p53 in cancer cells as an anti-cancer therapy [13, 14]. So far, a variety of compounds has been investigated to target mutant p53 [15, 16]. PRIMA-1 (p53-reactivation and induction of massive apoptosis-1), a low molecular weight compound (C_9_H_15_NO_3_), was discovered to restore the mutant p53 to the structure and function of wild-type p53, thus selectively killing cancer cells with mutant p53 [17]. PRIMA-1^met^ (APR-246), a methylated and more potent analog of PRIMA-1, has already advanced to a phase I/II clinic trial in hematologic malignancies and prostate cancer [18, 19]. It has been reported that PRIMA-1/PRIMA-1^met^ selectively restores the sequence-specific DNA binding region of mutated p53 via forming adducts with thiols and recovers its normal wild-type function to induce apoptosis in cancer cells [17, 20, 21]. However, some recent studies discovered that PRIMA-1/PRIMA-1^met^ also has inhibitory effect on cancer cells without p53 mutation [20, 22–25].

In this study, we aim to screen the effect of PRIMA-1^met^ on different CRC cell lines, in order to gain a deep and comprehensive understanding for the potential of PRIMA-1^met^ as an anti-cancer agent especially for CRC patients. We demonstrated that PRIMA-1^met^ inhibited CRC cells growth independently of p53 status. PRIMA-1^met^ induced robust apoptosis preferably in p53 mutant CRC cell lines, which was mediated through upregulated expression of pro-apoptotic Noxa. In addition, PRIMA-1^met^ also exerted effects on inhibition of proliferation, migration and colony formation in a p53-independent manner. PRIMA-1^met^ impeded CRC tumor progression in xenograft mouse model.

## RESULTS

### PRIMA-1^met^ inhibited CRC cell proliferation in a dose-dependent manner

**Figure 1:**
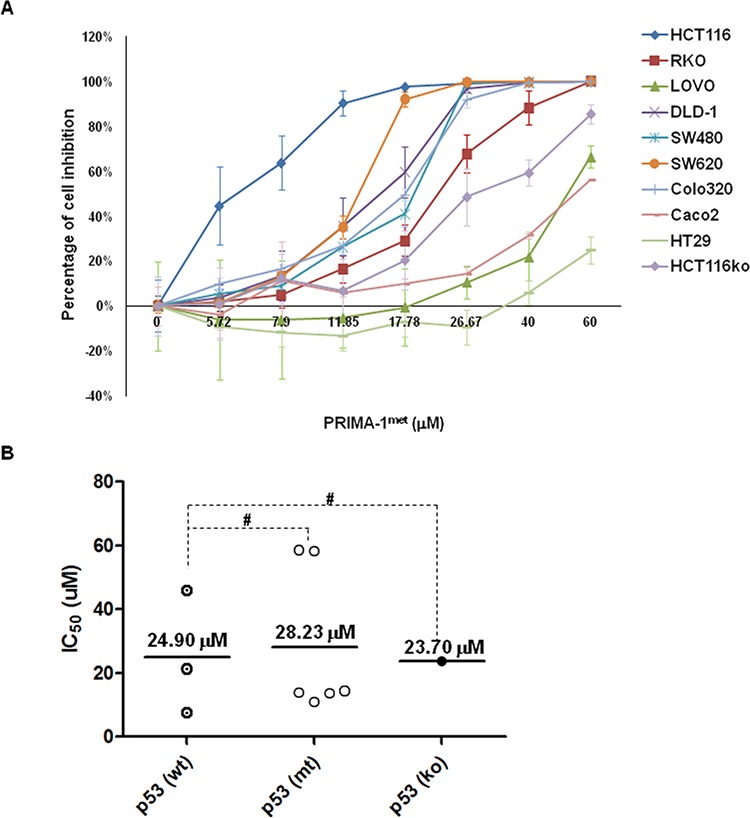
PRIMA-1^met^ inhibited cell proliferation in CRC cell lines A panel of CRC cell lines was treated with either DMSO control or PRIMA-1^met^ at indicated dosages for 48 hours, followed by CTG assays. The percentage of cell growth inhibition was normalized with respective DMSO control. **B.** IC_50_ of PRIMA-1^met^ in each cell line was calculated with CalcuSyn (Biosoft, Cambridge, UK) according to the fraction of cell inhibition. Dot plots were constructed and divided into 3 groups with wild type (wt) p53, mutant (mt) p53 and p53 knockout (ko) cell line, respectively. ^#^*p* > 0.05. All the experiments were triplicated and error bars represented standard error of mean (SEM).

We first investigated the effects of PRIMA-1^met^ on the growth of 10 human CRC cell lines with different p53 status. The results from the CTG assay showed a gradual decrease in cell proliferation along with progressive increases in the concentrations of PRIMA-1^met^ for 48 hours (Table 1 and Figure 1A and 1B). The IC_50_ and p53 status of these CRC cell lines were summarized in Table1. The inhibitory effect on cell growth induced by PRIMA-1^met^ was in a dose-dependent manner in all CRC cell lines. However, HT29 cell line exhibited more resistance to PRIMA-1^met^ than the others (Figure 1A). The means of IC_50_ in p53^wt^ CRC cell lines, p53^mt^ CRC cell lines, and p53^ko^ CRC cell lines were 24.90 μM (ranging from 7.5 to 45.9 μM), 28.23 μM (ranging from 10.9 to 58.6 μM) and 23.70 μM, respectively. Surprisingly, we did not observe significant difference in the IC50 values among the 3 groups of cell lines with distinct p53 status (Figure 1B, *p* > 0.05).

**Table 1:** The list of CRC cell lines with different p53 status and their IC_50_ values of PRIMA-1^met^

Cell lines	p53 status	IC_50_ (μM)
HCT116	wild type	7.5
RKO	wild type	21.3
LOVO	wild type	45.9
DLD-1	S241F, C > T	14.3
SW480	R273H, G > A	13.6
SW620	R273H, G > A	10.9
Colo320	R248W, C > T	13.8
Caco2	E204, G > T	58.6
HT29	R273H, G > A	58.2
HCT116^KO^	knock out	23.7

### PRIMA-1^met^ preferably induced robust apoptosis in CRC cells with mutant p53

**Figure 2:**
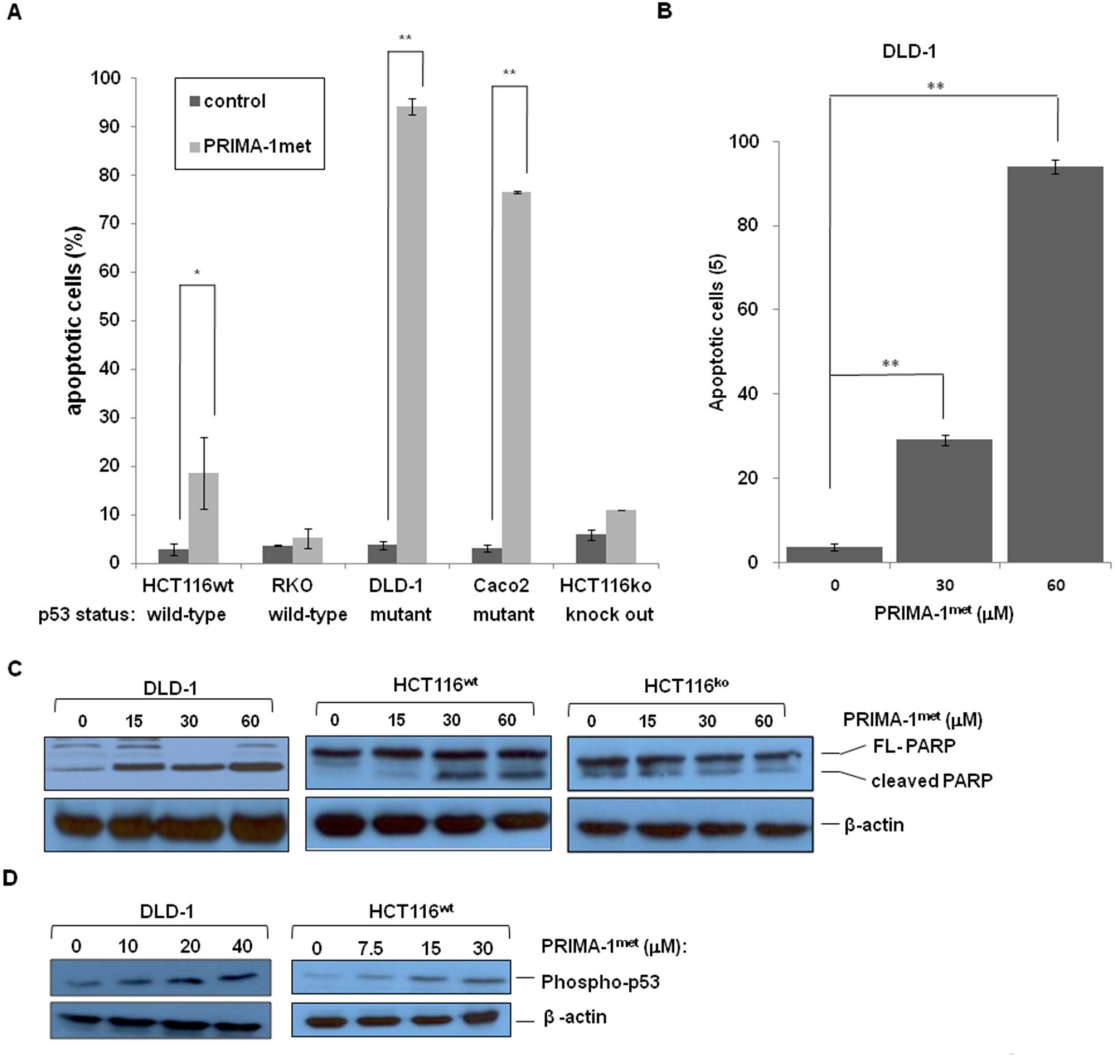
PRIMA-1^met^ induced apoptosis in CRC cell lines **A.** Apoptosis of different CRC cell lines was induced by PRIMA-1^met^. After treated with PRIMA-1^met^ for 48 hours, cell apoptosis was estimated by flow cytometric analysis. **B.** DLD-1 cells were treated with PRIMA-1^met^ at different concentrations, then followed by FACS analysis of apoptosis. In A. and B. *N* = 2, error bars show SEM. *p < 0.05; **p < 0.01. **C.** PARP cleavage was detected by western blotting analysis after treated with PRIMA-1^met^ at different concentrations for 24 hours. **D.** Phosphorylation of p53 level was determined after PRIMA-1^met^ treatment.

To investigate whether PRIMA-1^met^ induced apoptosis in CRC cell lines, we chose 2 cell lines with wild-type p53 (HCT116^wt^, RKO), 2 cell lines with mutant p53 (DLD-1, Caco2), and 1 cell line knocked out p53 (HCT116^ko^). As shown in Figure 2A, PRIMA-1^met^ treatment induced apoptosis in two CRC cell lines carrying mutant p53, and showed a dose-dependent effect in DLD-1 cell line (Figure 2B). Interestingly, PRIMA-1^met^ treatment also induced moderate apoptotic response in HCT116^wt^, in contrast, another p53 wild-type cell line RKO and p53-null HCT116 appeared to be resistant to PRIAM-1^met^ treatment (Figure 2A). Western blotting analysis revealed an increase of PARP cleavage in DLD-1 and HCT116^wt^ cell lines (Figure 2C). In agreement with the results from apoptosis assays by FACS analysis, an increase of PARP cleavage was not observed in p53-null HCT116 cells after PRIAM-1^met^ treatment (Figure 2C). Apoptotic induction of PRIMA-1^met^ was not only p53 dependent, but also related to p53 activation, because high expression of phospho-p53 (Ser15) could be detected after PRIMA-1^met^ treatment (Figure 2D). These results suggest that, in general, PRIMA-1^met^ induced-apoptosis is more prominent in CRC cells with mutant p53.

### PRIMA-1^met^ increased expression of Noxa, but not PUMA

**Figure 3:**
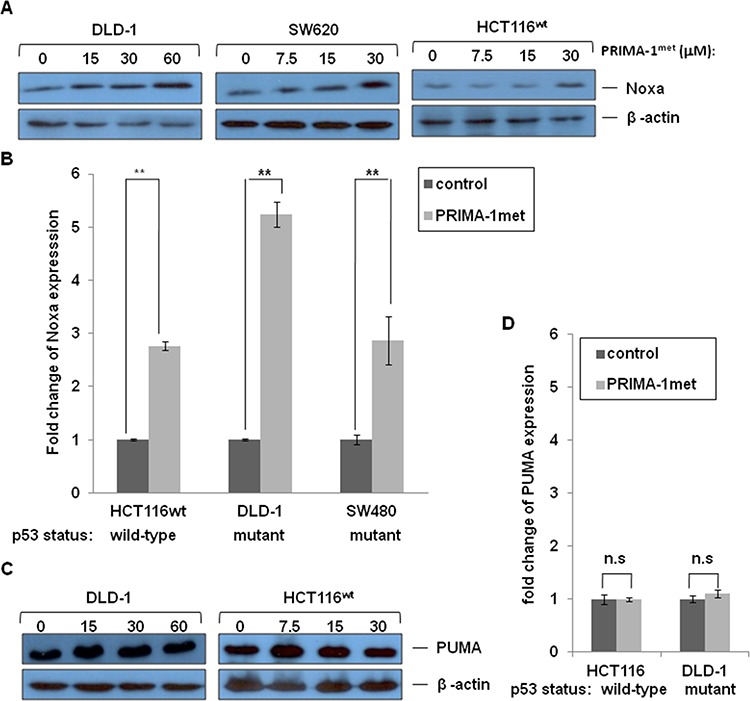
The effects of PRIMA-1^met^ on pro-apoptotic molecules Noxa and PUMA DLD-1, SW620 and HCT116^wt^ cells were treated with different concentrations of PRIMA-1^met^ for 24 hours. Cells then were harvested for analysis of Noxa protein levels **A.** and mRNA levels **B.** DLD-1 and HCT116^wt^ cells were treated with different concentrations of PRIMA-1^met^ for 24 hours. Cells then were harvested for analysis of PUMA protein levels **C.** and mRNA levels **D.** Expression of Noxa and PUMA genes was quantified using real-time RT-PCR on mRNA level. *N* = 2, error bars show SEM. *p < 0.05; **p < 0.01. n.s.: not significant, *p* > 0.05.

Under cellular stress, wild type (wt) p53 up-regulates its target proteins to promote apoptosis. Pro-apoptotic Bcl-2 (B-cell lymphoma-2) family members, such as Noxa and PUMA, are important target proteins [25, 31]. We performed western blotting analysis and qRT-PCR to evaluate expression of Noxa and PUMA. Cells were treated with PRIAM-1^met^ that resulted in strong expression of Noxa in p53 mutant DLD-1 and SW620 cell lines and p53 wild-type HCT-116 cell line (Figure 3A and 3B). However, the highest fold increase of expression of Noxa mRNA in DLD-1 cell line occurred concomitantly with the largest percentage of apoptosis observed in DLD-1 cells after PRIMA-1^met^ treatment (Figure 2A and Figure 3B). In contrast, PRIAM-1^met^ treatment did not induce expression of another pro-apoptotic gene, PUMA, on either RNA level or protein level (Figure 3C and 3D). Taken together, these data indicated that Noxa played a pivotal role in the activity of PRIMA-1^met^.

### PRIMA-1^met^ induced apoptosis in CRC cell lines with mutant p53 is mediated through Noxa

**Figure 4:**
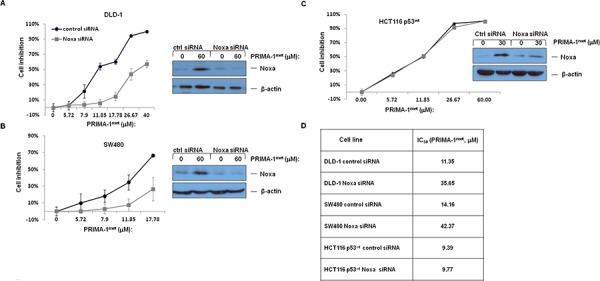
The impact of PRIMA-1^met^ on cell proliferation after treatment of si-Noxa in DLD-1 **A.** SW480 **B.** and HCT116wt **C.** *N* = 3, error bars show SEM. Insert pictures at the left showing alterations in Noxa protein after transfection with Control siRNA or siRNA specifically targeting Noxa (si-Noxa) for 24 hours with or without PRIMA-1^met^. **D.** IC_50_ values of PRIMA-1^met^ in CRC cell lines transfected with either control siRNA or Noxa specific siRNA.

To further clarify the role of Noxa in PRIMA-1^met^ induced-apoptosis, we knocked down Noxa gene in DLD-1, SW480 and HCT116^wt^ cell lines. Western blotting analysis confirmed the reduced Noxa proten level upon Noxa specific siRNA treatment in these 3 cell lines (inserted pictures in Figure 4A, 4B and 4C). We observed that the inhibitory effect on two mutant p53 cell lines, DLD-1 and SW480, were significantly reduced after knocking down Noxa, whereas knocking down Noxa in wt p53 HCT1116 failed to produce any noticeable impact on the IC_50_ (Figure 4A, 4B, 4C and 4D).

### PRIMA-1^met^ inhibited cell migration, invasion and colony formation in CRC cells

**Figure 5:**
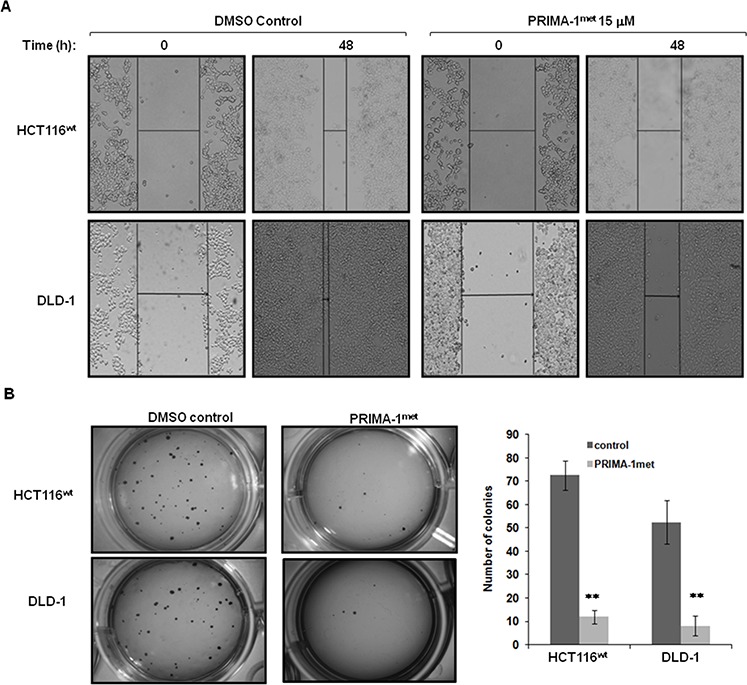
PRIMA-1^met^ suppressed CRC cell migration and colony formation **A.** DLD-1 and HCT116^wt^ cells were seeded into a six-well tissue culture dish and allowed to grow to 90% confluency in complete medium. Cell monolayers were wounded by a plastic tip (1 mm) that touched the plate and put back into the incubator for 48 hours. Cells were monitored under a microscope equipped with a camera (Olympus, Japan). Each experiment was repeated three times and representative images were shown. **B.** To assay colony formation, 2 × 10^3^ DLD-1 and HCT116^wt^ cells were plated in triplicates into 12-well cell culture plates with DMSO or PRIMA-1^met^ in the soft agar. After 21 days, the number of foci on each dish was counted. Colonies with more than 20 cells were scored as positive. Each experiment was repeated three times. The bar figure represented the data of mean ± S.E. of three determinations. **p < 0.001.

To explore the effect of PRIMA-1^met^ on the migration and invasion of CRC cells, a wound healing assay was employed to compare CRC cells treated with either DMSO or PRIMA-1^met^. As shown in Figure 5, relative to DMSO treated controls, wound healing was significantly impaired in HCT116^wt^ and DLD-1 cells treated with PRIMA-1^met^. These findings strongly suggest that PRIMA-1^met^ inhibits motility signalling in CRC cells. Morphological transformation of cell colonies *in vitro* has a high correlation with carcinogenesis *in vivo*. We conducted soft agar assay to assess the impact of PRIMA-1^met^ on colony formation of CRC cells. After treated with PRIMA-1^met^ for 48 hours, cells were seeded in soft agar and incubated for 21 days. We found that PRIMA-1^met^ greatly reduced the number and size of HCT116^wt^ and DLD-1 cell colonies (Figure 5B). Altogether, the *in vitro* migration and soft agar assay indicated that the ability of PRIMA-1^met^ to inhibition cell migration and colony formation of CRC cells also contributed to its anti-cancer activity, independent of p53 status.

### PRIMA-1^met^ suppressed tumor growth in xenograft mouse CRC model

**Figure 6:**
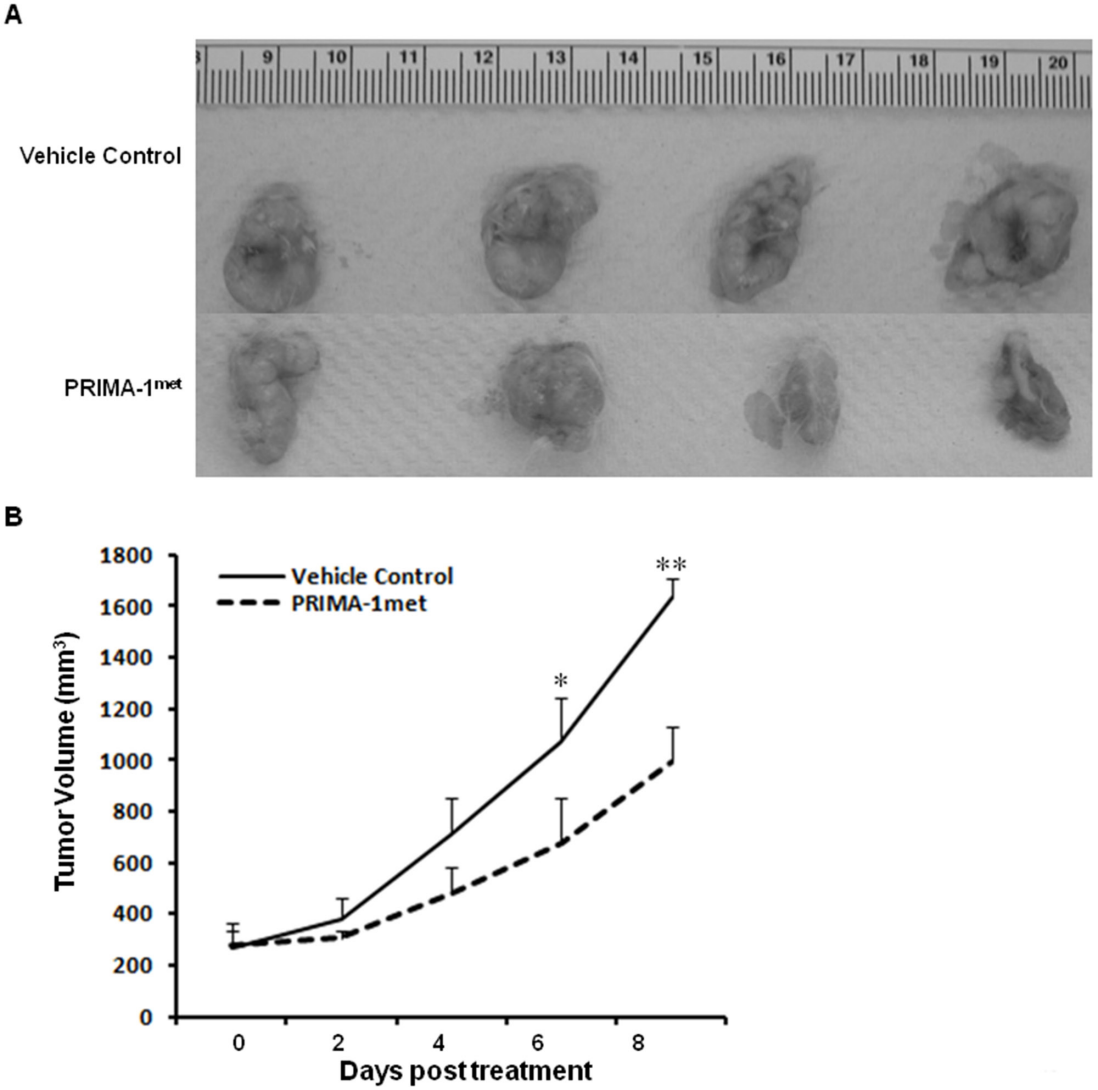
PRIMA-1^met^ significantly suppressed CRC xenograft growth **A.** The images of DLD-1 tumors from mice treated with vehicle control versus PRIMA-1^met^ (*n* = 4 for each group). **B.** PRIMA-1^met^ significantly suppressed tumor growth. *p < 0.05; **p < 0.01.

To validate the anti-tumorigenic potential of PRIMA-1^met^ *in vivo*, we utilised a xenograft CRC mouse model by subcutaneously injecting DLD-1 cells into female NOD/SCID mice. After the mice developed palpable tumor masses, we began PRIMA-1^met^ treatment (intraperitoneal injection, 50 mg/kg) on the mice daily for 8 days. Tumor growth during the treatment was monitored by caliper measurement every other day. At day 8 of post treatment, the mice were euthanized, and the tumors were removed, and photographed. We observed a substantial reduction in tumor growth in the PRIMA-1^met^ treated group in comparison with the vehicle control treated group (Figure 6A). The PRIMA-1^met^ treatment group presented with tumor sizes substantially smaller than those of the controls (Figure 6B), indicating the efficacy of the PRIMA-1^met^ in CRC regression. It is important to note that during the treatment, the mice showed tolerance to PRIMA-1^met^ injections and maintained normal activities and no loss of weight were recorded (data not shown).

## DISCUSSION

In this study, we shed new light on the effect of PRIMA-1^met^ on CRC. Our results showed that PRIMA-1^met^ inhibited CRC cell growth independent of p53 status in a dose-dependent manner, but preferably induced robust apoptosis in mutant p53 CRC cells. We further uncovered the molecular mechanism underlying PRIMA-1^met^ induced-apoptosis through up-regulation of pro-apoptotic protein Noxa, leading to activation of cleavage of PARP. These results were consistent with previous studies that demonstrated the ability of PRIMA-1^met^ to restore the mutant p53 to wild-type p53 properties in other cancers [15, 17, 20, 21, 25]. Reduction of Noxa expression by siRNA accentuated the inhibitive effect of PRIMA-1^met^ and increased IC_50_ of PRIMA-1^met^ in DLD-1 and SW480 cell lines. These results suggest that upregulation of Noxa plays an influential role in the mediation of cell proliferation and apoptosis in CRC cells treated with PRIMA-1^met^. The importance of Noxa also has been noted in PRIMA-1^met^ treated multiple myeloma cells [25].

Most studies reported that PRIMA-1^met^ targets mutant p53 proteins [17, 32, 33]. Interestingly, in this study, CRC cell lines with wild-type p53 or without p53 responded to PRIMA-1^met^ treatment. Some IC_50_ values of these cell lines with wild-type p53 were even less than those carrying mutant p53. However, different mechanisms might contribute to the effectiveness of PRIMA-1^met^ observed in these two types of CRC cells. Unlike in p53 mutant CRC cells, PRIMA-1^met^ mainly induced cytostasis, but to less extent apoptosis, in CRC cells with wt p53 or p53 null. The percentage of apoptotic cells did not increased significantly after treated with PRIMA-1^met^ in cell lines RKO (p53 wt) and HCT116^ko^. In addition, knocking down Noxa gene reduced PRIMA-1^met^ sensitivity in mutant p53 CRC cells, but it didn't significantly decrease PRIMA-1^met^ sensitivity in HCT116^wt^ cell line. Taken together, these results support a model where although PRIMA-1^met^ inhibits proliferation of CRC cells independently on p53 status. Nevertheless, PRIMA-1^met^ mainly induces cytostasis in CRC cells with wt p53 or p53 null, whereas PRIMA-1^met^ promotes apoptosis in CRC cells with mutant p53. More importantly, PRIMA-1^met^ treatment significantly reduced tumor growth in our mouse model and showed no noticeable toxicity to the mice.

In conclusion, in our study PRIMA-1^met^ inhibits CRC cells growth, colonies formation, proliferation and migration in a p53-independent manner. However, the ability of to induce apoptosis appears specific in CRC cells with mutant p53. Our *in vitro* and *in vivo* results suggest that PRIMA-1^met^ is an attractive candidate for further investigation as a potential anti-cancer agent in clinical trials for patients with CRC. However, we also acknowledge that the work is limited by studying CRC lines. A deeper understanding of the molecular mechanisms of anti-tumorigenic activity of PRIMA-1^met^ will allow for precise treatment regimens and the possibility of co-treatment with other therapeutics to greatly improve the outcome of CRC patients.

## MATERIALS AND METHODS

### Cell lines and drugs

A panel of colorectal cancer cell lines with different p53 status was used in this study. The details of these cell lines were: LOVO (wild-type p53), RKO (wild-type p53), SW480 (mutant p53-R237H), SW620 (mutant p53-R237H), HT29 (mutant p53-R237H), Colo320 (mutant p53-R248W) and Caco2 (mutant p53-E204X). These cells were provided by Dr. Motomi Osato (Cancer Science Institute of Singapore, National University of Singapore). HCT116 (wild-type p53+/+, HCT116^wt^) and HCT116 null (p53−/−, HCT116^ko^) cell lines were gifts from Dr. Jimmy Chao (Bioprocessing Technology Institute, A*Star, Singapore). DLD-1 (mutant p53-S241F) cell line was obtained in house. Cells were grown in DMEM (Dulbecco's Modified Eagle's Medium) (Invitrogen, Carlsbad, CA) with 10% inactivated fetal bovine serum (FBS, JRH Bioscience Inc, Lenexa, KS) and 1% penicillin/streptomycin (Invitrogen, Carlsbad, CA), in a humid incubator with 5% CO_2_ at 37°C. PRIMA-1^met^ (APR-246, Santa Cruz Biotechnology, Santa Cruz, CA) was dissolved in DMSO (Dimethyl sulfoxide) at a concentration of 50 mM and stored at −20°C. Working solutions were then diluted to appropriate concentrations in cell culture medium. Same amount of DMSO as control reagent was added in each experiment.

### Cell proliferation assay

After 24 hours of seeding at 5000 cells/well in 100 μl medium in a 96-well plate, cells were treated with PRIMA-1^met^ at concentrations from 5.72 μM to 60 μM, and incubated for 48 hours. CellTiter-Glo (CTG) Luminescent Cell Viability Assay (Promega, Madsion, WI) was used to measure the cell proliferation by quantitating the cell's ATP as previously reported [26]. The reading of DMSO control samples were set as 100% and the reading of other samples were calculated as relative proliferation to the DMSO control samples. Each experiment was in triplicate. The results were presented in percentage changes relative to their respective DMSO controls.

### Cell apoptosis assay

Cells were seeded on 6-well plates at a density of 0.2 × 10^6^ cells per well and treated with 30 μM, 60 μM PRIMA-1^met^, respectively, for 48 hours. Apoptosis assay was conducted with flow cytometric analysis of Annexin V-FITC (Invitrogen) and Sytox Blue (BD Biosciences, San Jose, CA) staining cells [27]. After suspending using trypsin and washing twice with cold PBS, cells were counted and adjusted at 0.5 - 1.0 × 10^6^ for each sample, and resuspended in 100 μl 1 × binding buffer. Each sample was added 1.5 μl Annexin V-FITC, and incubated on ice in dark for 20 minutes. Then after added 1 μl Sytox Blue and incubated at room temperature for 5 minutes in dark, each sample was mixed with 400 μl of 1 × binding buffer, filtered to a 5 ml BD Falcon tube (BD Biosciences), then to analyze by flow cytometry within 1 hour.

### Western blotting analysis

We plated cells at 2 × 10^6^ per 10cm dish. After 24 hours, cells were treated with DMSO control and different dose of PRIMA-1^met^ as indicated. After 24 hours, cells were lysed and analyzed by western blotting as described previously [28]. Primary antibodies used in this study were against p53 (sc-126, Santa Cruz Biotechnology), Actin (sc-1616, Santa Cruz Biotechnology), cleaved PARP (D214), Phospho-p53 (Ser15) and PUMA from Cell Signaling Technology (Danvers, MA), Noxa (EMD chemicals, Gibbstown, NJ). Secondary antibodies were goat-anti-rabbit IgG HRP (sc-2030, Santa Cruz Biotechnology) and goat-anti-mouse IgG HRP (sc-2031, Santa Cruz Biotechnology).

### Real-time quantitative reverse transcriptase-PCR (qRT-PCR)

All CRC cells were seeded in a 6-well plate at a density of 0.4 × 10^6^ cells/well to grow overnight. RKO, Colo320, DLD-1 and SW480 cells were treated with 60 μM PRIMA-1^met^ for 24 hours. HCT116^wt^ cells with 30 μM PRIMA-1^met^ and HCT116^ko^ cells with 120 μM PRIMA-1^met^ were incubated for 24 hours. RNA was isolated from cells using RNeasy mini kit (Qiagen, Valencia, CA). Typically, 1 μg of total RNA was used to generate cDNA by using SuperScript^®^ III RT (Invitrogen) with oligo-dT primer. qRT-PCR was performed using the Power SYBR^®^ Green PCR Master Mix as recommended by the manufacturer (Applied Biosystems, Carlsbad, CA). GAPDH was used as the internal control. SDS 2.2.1 software (Applied Biosystems) was used to perform relative quantification of target genes using the comparative C_T_ (ΔΔC_T_) method. The primer sequences were described as the following: Noxa (Forward: 5′-GCTGGAAGTCGAGTGTGCTA-3′, Reverse: 5′-CCTGAGCAGAAGAGTTTGGA-3′) and PUMA (Forward: 5′-ACGACCTCAACGCACAGTACG-3′, Reverse: 5′-TCCCATGA TGAGATTGTACAGGAC-3′) (Integrated DNA Technologies, Singapore).

### siRNA transfection

HCT116^wt^, DLD-1 and SW480 cells were seeded in 6-well plates 1 day for transfection at a density of 3 × 10^6^ cells/well. Mixture containing 110 pmoles of Noxa siRNA (sc-37305, Santa Cruz Biotechnology) or control siRNA (sc-36869, Santa Cruz Biotechnology), 200 μl of jetPRIME^®^ buffer (Polyplus-Transfection, Illkich, Franc) and 4 μl of jetPRIME^®^ *in vitro* siRNA transfection reagent (Polyplus-Transfection) was vortexed 10 seconds, spun down and incubated 10 minutes at room temperature. This transfection mixture was added to each well. After incubated for 24 hours, cells were harvested for further analysis.

### Wound healing assay

HCT116^wt^ and DLD-1 cells were seeded to four wells in 6-well plates at 1 × 10^6^ cells/well for duplication. After 3 days, cells were cultured to near confluence and scratched a wound through the centre of the well. Rinsed with PBS three times gently, two of these wells were replaced with 2 ml of media containing DMSO as control. The others were added with PRIMA-1^met^ at concentration of 15 μM. After taken pictures under microscope (10 × magnification), plates were transferred to humidified incubator at 37°C and incubated for 48 hours. Pictures were taken again.

### Soft agar assay

After seeded in 6-well plate at a density of 2 × 10^5^ cells/well overnight, HCT116^wt^ and were harvested and 2 × 10^3^ each cell line to mix with 3 ml top agar (containing PRIMA-1^met^ 10 μM, 20 μM, respectively) gently. The mixture was added to each of the duplicated wells in a 12-well plate with base agar (containing 0.75 ml 1 × DMEM+10% FCS and 0.75 ml 1% agar in each well) prepared before. The plate was incubated in humidified incubator at 37°C for 21 days, and then to stained with 0.5 ml of 0.005% crystal violet (Sigma-Aldrich, St. Louis, MO) for 1 hour. Cell colonies formation was observed under microscope (200× magnification).

### Xenograft mouse model

Female Nonobese diabetic/severe combined immunodeficiency (NOD/SCID) mice (17–20 g, 4–6 weeks old) were purchased from InVivos (Singapore). The preparation and injection of DLD-1 cells were followed the reported methods [29, 30]. In briefly, exponentially growing DLD-1 cells (4 × 10^6^) were subcutaneously injected into loose skin between the shoulder blades and left front leg of recipient mice. All treatments were started 14 days after the injection, when the mice had palpable tumors of an average size of approximately 200 mm3. PRIMA-1^met^ was dissolved in 1x PBS and administrated at 50 mg/kg/day by in intraperitoneal injection (I.P.) daily. Mice in vehicle control group were treated with 1 X PBS daily. Treatments lasted for 14 days. Each group was comprised of 10 mice.

The length (L) and width (W) of the tumor were measured with callipers, and tumor volume (TV) was calculated as TV = (L × W^2^)/2. The protocol was reviewed and approved by Institutional Animal Care and Use Committee in compliance to the guidelines on the care and use of animals for scientific purpose.

### Statistical analysis

The statistical analysis was performed by SPSS version 13.0. Values were expressed as mean ± standard error of the mean (SEM). Comparison between groups of data was made using one-way analysis of variance (ANOVA). Tumour volume reduction of the treatment groups was compared to the vehicle control group by Student's *t*-test. *p* < 0.05 was considered statistically significant.
